# The complete genomic sequence of a novel cold-adapted bacterium, *Planococcus maritimus* Y42, isolated from crude oil-contaminated soil

**DOI:** 10.1186/s40793-018-0328-9

**Published:** 2018-10-10

**Authors:** Ruiqi Yang, Guangxiu Liu, Tuo Chen, Wei Zhang, Gaosen Zhang, Sijing Chang

**Affiliations:** 1 Key Laboratory of Desert and Desertification, Northwest Institute of Eco-Environment and Resources, Chinese Academy of Sciences, Lanzhou, 730000 China; 2 Key Laboratory of Extreme Environmental Microbial Resources and Engineering, Lanzhou, 730000 Gansu Province China; 3 State Key Laboratory of Cryospheric Sciences, Northwest Institute of Eco-Environment and Resources, Chinese Academy of Sciences, Lanzhou, 730000 China; 4 University of Chinese Academy of Sciences, No.19A Yuquan Road, Beijing, 100049 China

**Keywords:** *Planococcus maritimus*, Qaidam Basin, Crude oil, Degradation, Genome

## Abstract

*Planococcus maritimus* Y42, isolated from the petroleum-contaminated soil of the Qaidam Basin, can use crude oil as its sole source of carbon and energy at 20 °C. The genome of *P. maritimus* strain Y42 has been sequenced to provide information on its properties. Genomic analysis shows that the genome of strain Y42 contains one circular DNA chromosome with a size of 3,718,896 bp and a GC content of 48.8%, and three plasmids (329,482; 89,073; and 12,282 bp). Although the strain Y42 did not show a remarkably higher ability in degrading crude oil than other oil-degrading bacteria, the existence of strain Y42 played a significant role to reducing the overall environmental impact as an indigenous oil-degrading bacterium. In addition, genome annotation revealed that strain Y42 has many genes responsible for hydrocarbon degradation. Structural features of the genomes might provide a competitive edge for *P. maritimus* strain Y42 to survive in oil-polluted environments and be worthy of further study in oil degradation for the recovery of crude oil-polluted environments.

## Introduction

Oil spills occur frequently and pose a severe hazard to pristine ecological conditions [1, 2]. On account of the difficulty in degrading crude oil, the pollutant remains in the environment to contaminate ground water and air, affect crop growth and endanger human health [3, 4]. Bioremediation is currently recognized as the preferred strategy to utilize biological activities to rapidly eliminate hydrocarbon pollutants [5]. Many microorganisms, especially bacteria, have been found to participate in the process of biodegradation in contaminated environments [6, 7].

*Planococcus*, as a psychrotolerant and halotolerant bacterium, was also reported as having the ability to degrade crude oil [8–10]. For example, a cultured *Planococcus* sp. strain S5 was described to be able to grow on salicylate or benzoate [11], and *Planococcus alkanoclasticus* was capable of degrading linear alkanes [9]. Meanwhile, most of the *Planococcus* bacteria have showed the ability to withstand heavy metals, produce surfactants and adapt to cold and/or saline environments [12–14]. Because of the above properties, *Planococcus* exhibited a potential capability in the bioremediation of extremely contaminated environments. Although many studies have reported the genomic backgrounds of *Planococcus* strains, oil biodegradation mechanisms in *Planococcus* have rarely been discussed. In the present study, we isolated a *Planococcus* strain from the oil-contaminated soils in the Qinghai-Tibetan Plateau. Our aims were to characterize the genome of this oil-degrading strain and to further seek responsible strategies associated with oil degradation in low-temperature environments.

## Organism information

### Classification and features

**Fig. 1:**
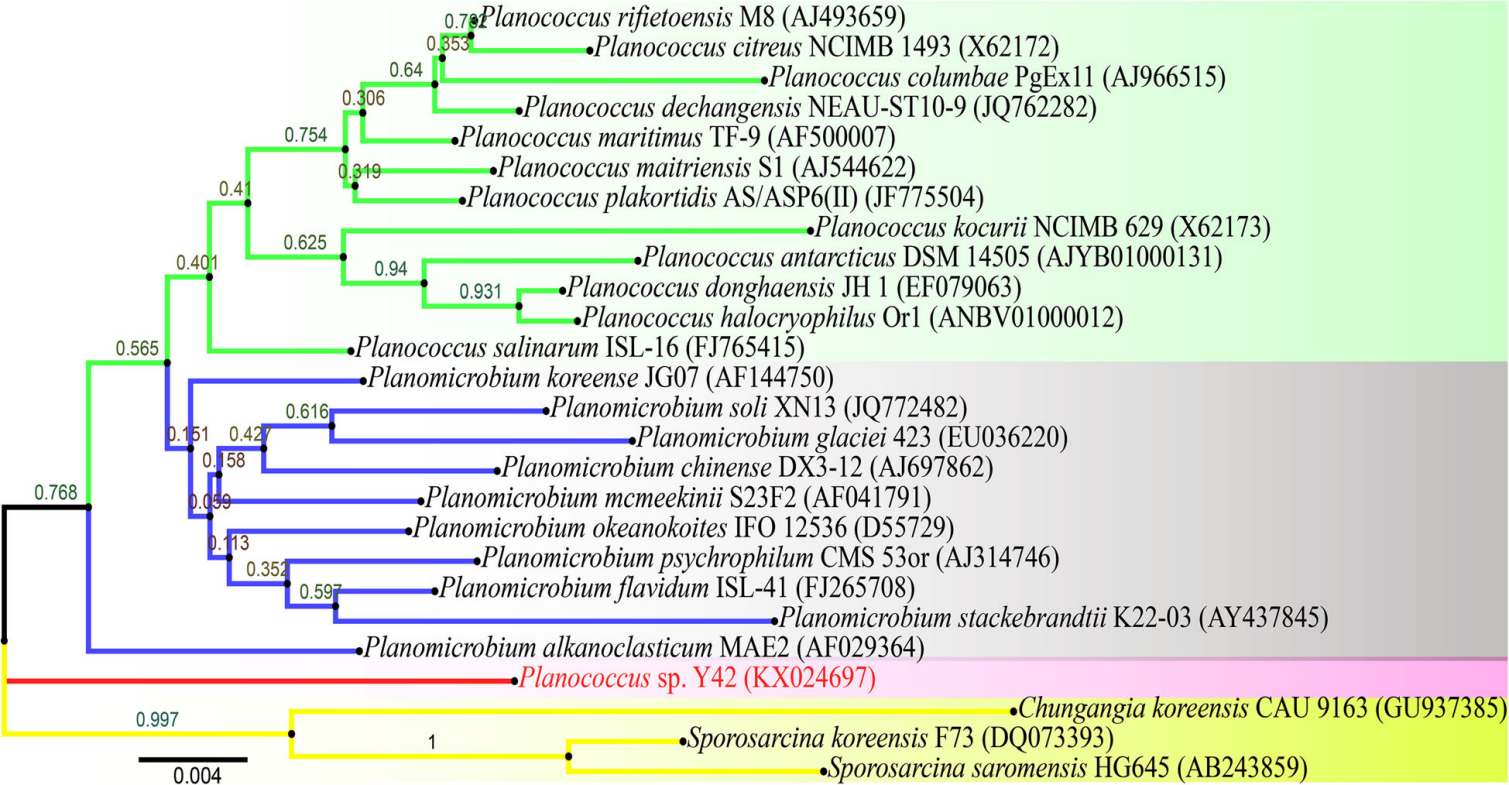
Phylogenetic tree of *P. maritimus* Y42 between known species of *Planococcus* genus. The phylogenetic tree constructed from the 16S rRNA sequence together with other *Planococcus* homologs using MEGA 6.0 software suite. The evolutionary history was inferred by using Neighbor-joining method based on model

In this experiment, a novel cold-adapted strain Y42 was isolated from oil-contaminated soils in the Lenghu oil field, which is located in the northern margin of the Qaidam Basin (93.34°E, 38.71°N). The molecular identification of the strain was performed using the primers 27F and 1492R to amplify and sequence the 16S rRNA gene [15]. Phylogenetic analysis based on 16S rRNA gene sequence similarity showed that strain Y42 was closely related to members of the genus *Planococcus* (*Planococcus maritimus* (97%)). The strain Y42 was thus recognized as a potential new member of *Planococcus* (Fig. 1).

**Fig. 2:**
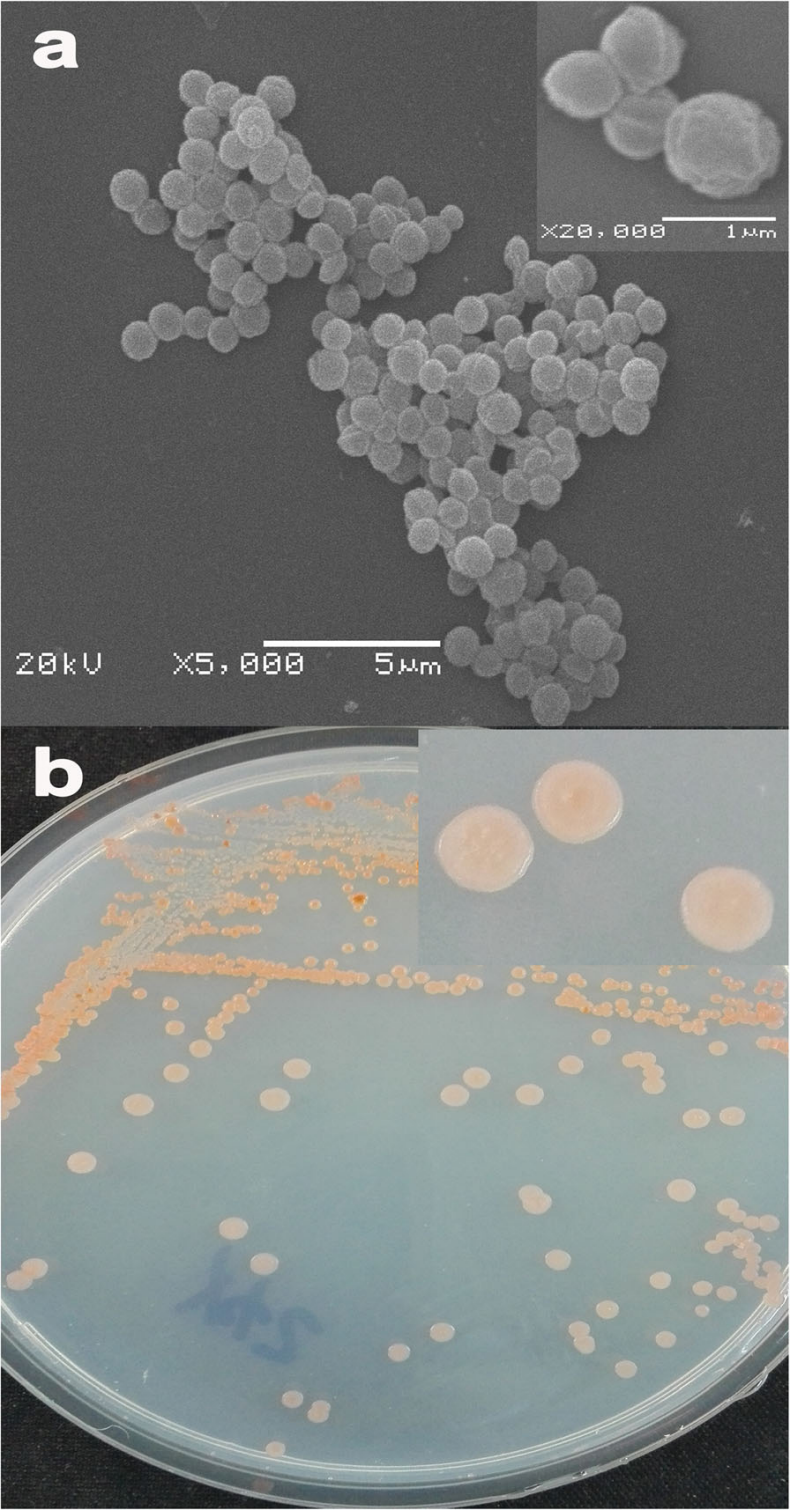
Scanning electron microscope (**a**) and Colony morphology on the 216 L plate (**b**) of *P. maritimus* Y42

The strain Y42 was able to grow at moderately low temperatures, and many members of the genus *Planococcus* had been predominantly isolated from frozen and/or saline environments [16]. Cell micrographs were obtained by using a scanning electron microscope (SEM) on cells grown in LB medium. Cells of strain Y42 were coccoid, typically 0.7–1 m in diameter, and diplococci were observed, along with cell division septa (Fig. 2a). Colony morphology was determined on LB plates following 3–5 days of growth at 25 °C, which resulted in the formation of orange, round, umbonate colonies (Fig. 2b). Additional characteristics of *P. maritimus* Y42 are shown in Table 1.

**Table 1:** Classification and general features of *P. maritimus* Y42

MIGS ID	Property	Term	Evidence code
	Classification	Domain Bacteria	TAS [42]
		Phylum *Firmicutes*	TAS [43]
		Class *Bacilli*	TAS [44, 45]
		Order *Bacillales*	TAS [46, 47]
		Family *Planococcaceae*	TAS [46, 48]
		Genus *Planococcus*	TAS [46, 49]
		Species *Planococcus*	
		Strain Y42	
	Gram stain	Positive	TAS [50]
	Cell shape	Coccoid	IDA
	Motility	Motile	TAS [50]
	Sporulation	Non-sporulating	TAS [50]
	Temperature range	4–30 °C	IDA
	Optimum temperature	25 °C	IDA
	pH range; Optimum	6–9; 7.5;	IDA
	Carbon source	Yeast extract	IDA
MIGS-6	Habitat	Frozen soil	IDA
MIGS-6.3	Salinity	< 15% NaCl (*w*/*v*)	TAS [50]
MIGS-22	Oxygen requirement	Aerobic	NAS
MIGS-15	Biotic relationship	Free-living	IDA
MIGS-14	Pathogenicity	Non-pathogen	NAS
MIGS-4	Geographic location	China: Qaidam Basin, Lenghu area	IDA
MIGS-5	Sample collection	2015	IDA
MIGS-4.1	Latitude	+ 38.71 (38°43′10.11″)	NAS
MIGS-4.2	Longitude	+ 93.34 (93°20′30.1″)	NAS
MIGS-4.4	Altitude	2789 m	NAS

^a^Evidence codes – *IDA* Inferred from Direct Assay, *TAS* Traceable Author Statement (i.e., a direct report exists in the literature), *NAS* Non-traceable. Author Statement (i.e., not directly observed for the living, isolated sample, but based on a generally accepted property for the species, or anecdotal evidence). These evidence codes are from the Gene Ontology project

**Fig. 3:**
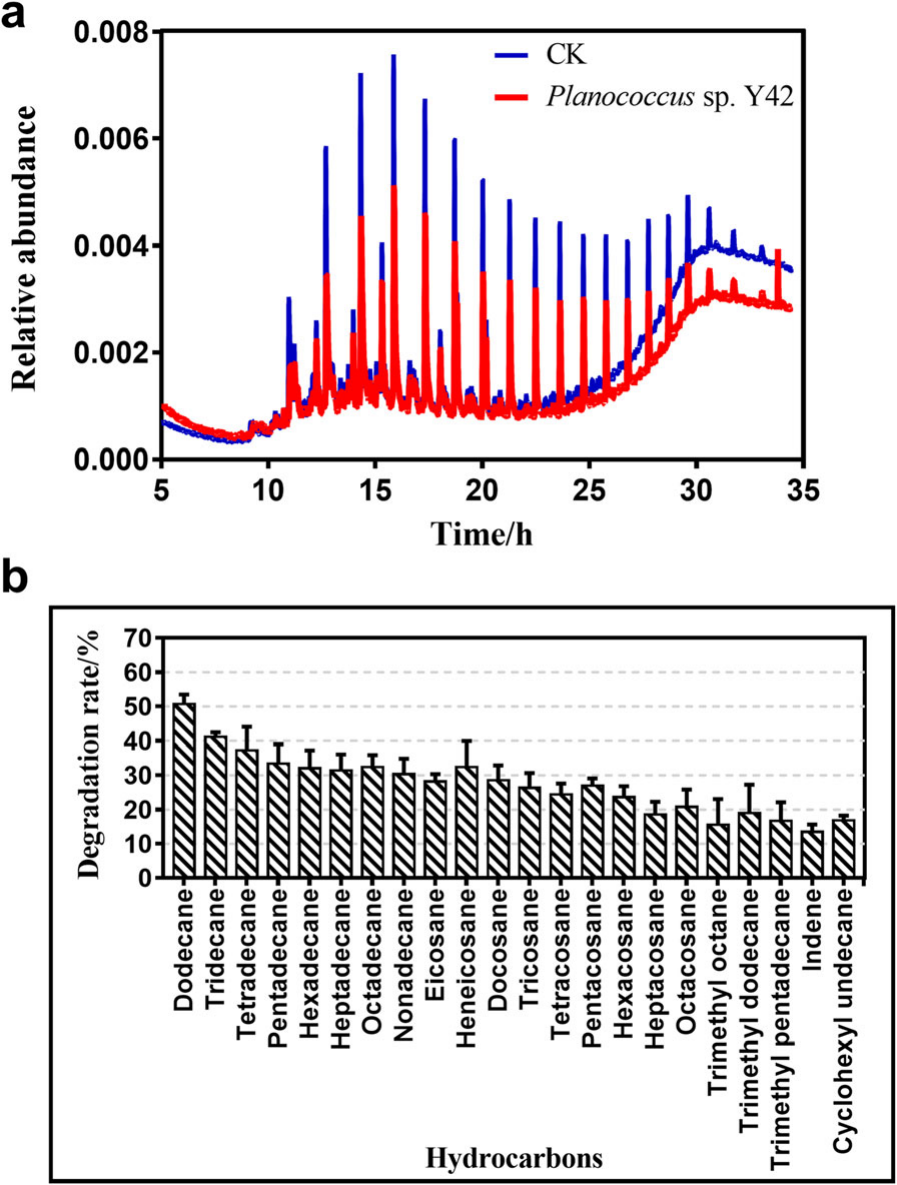
The gas chromatograms of crude oil after degradation by *P. maritimus* Y42. **a** Total ion currents (TIC) of gas chromatography-mass spectrometer (GC-MS) monitoring the component variations of the residual crude oil (evaporated residue) before (the blue) and after (the red) incubation with strain Y42. **b** Degradation rates of the hydrocarbon components in evaporated crude oil by strain Y42 after 10 days of incubation at 20 °C

Crude oil-degrading characterization of strain Y42 was completed under specified growth conditions with crude oil as the sole carbon source by using a gas chromatography-mass spectrometry (GC-MS) method. The strain Y42 was cultured with MM medium (3.5 g of MgCl_2_, 1.0 g of NH_4_NO_3_, 0.35 g of KCl, 0.05 g of CaCl_2_, 1.0 g of KH_2_PO_4_, 1.0 g of K_2_HPO_4_, 0.01 g of FeCl_3_, 0.08 g of KBr, and 24 mg of SrCl_2_·6H_2_O, pH 7.5) with crude oil as a carbon source and incubated at 20 °C for 10 d [17]. A parallel experiment without inoculation was used as the control. The remaining oil from the cultures was extracted with 15 mL of hexane in a separating funnel at room temperature, and anhydrous Na_2_SO_4_ was then added to remove residual water. Ultimately, the extracted oil was analysed using a GC-MS method [18]. For GC-MS analysis, one microliter of the filtered solution was injected into a quartz capillary column (DB-WAX, 30 m × 0.25 mm × 0.25 μm). The total area of a detected individual hydrocarbon peak was defined as its hydrocarbon concentration in crude oil. The degradation rate of the components of crude oil was determined according to the following equation: η = (1-n1/n2) × 100%, where η, n_1_ and n_2_ are the degradation rate of the components of crude oil, the peak area of the components of crude oil remaining in the samples, and the peak area of the components of crude oil in the controls, respectively [19]. The chromatograms revealed that the concentrations of the components of crude oil, including *n*-alkanes, branched alkanes, cyclanes, and aromatic hydrocarbons, were lower in the sample treated with the strain *P. maritimus* Y42 than the abiotic control sample (Fig. 3a). After incubation for 10 days at 20 °C, the preferred degradation occurred in short-chain *n*-alkanes ranging from C_12_ to C_18_, C_12_ was particular decomposed, by approximately 50%. Meanwhile, the other straight-chain alkanes and aromatic hydrocarbons were decomposed by 20–30% (Fig. 3b). The strain Y42 did not show a remarkably higher ability to degrade different components of crude oil than other strains such as *Bacillus* [20, 21], *Pseudomonas* [22, 23], *Rhodococcus* [24] and etceteras. Even so, as an indigenous oil-degrading bacterium, the existence of the *P. maritimus* strain Y42 played a significant role in reducing overall environmental impact of the oil [25] and greatly enriched microbial community structures in the oil-contaminated soils in low-temperature environments [26].

## Genome sequencing information

### Genome project history

This organism was selected for sequencing based on its phylogenetic position and its ability to degrade crude oil. The genome project was deposited in the genome online database [27] and the complete genome sequence was available in GenBank (NCBI-Genome). Sequencing, finishing and annotation were performed by the DOE Joint Genome Institute (JGI). A summary of the project information was provided in Table 2.

**Table 2:** Project information of the whole genome sequence of *P. maritimus* Y42

MIGS ID	Property	Term
MIGS-31	Finishing quality	Finished
MIGS-28	Libraries used	Paired-end and PacBio
MIGS-29	Sequencing platforms	Illumina Hiseq 2000 and PacBio
MIGS-31.2	Fold coverage	PacBio: 300×
MIGS-30	Assemblers	SPAdes v. 3.5.0, HGAP
MIGS-32	Gene calling method	Glimmer 3.02
	Locus Tag	B0X71
	GenBank ID	CP019640.1-CP019643.1
	GenBank Date of Release	April 14, 2017
	GOLD ID	Gp0209326
	BIOPROJECT	PRJNA371518
MIGS-13	Source Material Identifier	Y42
	Project relevance	Biodegrading

### Growth conditions and genomic DNA preparation

*P. maritimus* strain Y42 was inoculated into LB liquid medium and grown on a gyratory shaker (200 rpm) at 20 °C for 96 h. Genomic DNA of the strain was extracted using the Bacterial Genomic DNA Extraction Kit (AxyPrep) as per its operation instruction.

### Genome sequencing and assembly

The complete genome sequence of *P. maritimus* strain Y42 was generated by combined Illumina MiSeq with PacBio platform [28]. The reads generated with Illumina MiSeq platform were denovo assembled using Newbler (version 2.8). The sub-reads generated from PacBio platform were de novo assembled using Hierarchical Genome Assembly Process (HGAP) [29]. Gaps between contigs were closed by using the SPAdes-3.5.0. This whole genome project (Bioproject ID: PRJNA371518) has been registered and assembled sequence data submitted at NCBI GenBank under the accession no. CP019640.1-CP019643.1. And this finished genome was deposited in IMG database with the Project ID: Gp0209326.

### Genome annotation

The completed genomic sequence was predicted using the Glimmer software 3.0 [30]. tRNA genes were predicted using tRNAscan-SE 1.3.1 [31] and rRNA genes were identified using Barrnap 0.4.2 [32]. The rest of the non-coding rRNA genes were predicted by using BLASTp against databases NCBI-NR database (http://www.ncbi.nlm.nih.gov/) and genes function annotations were assigned by the COG database (http://www.ncbi.nlm.nih.gov/COG/).

## Genome properties

**Fig. 4:**
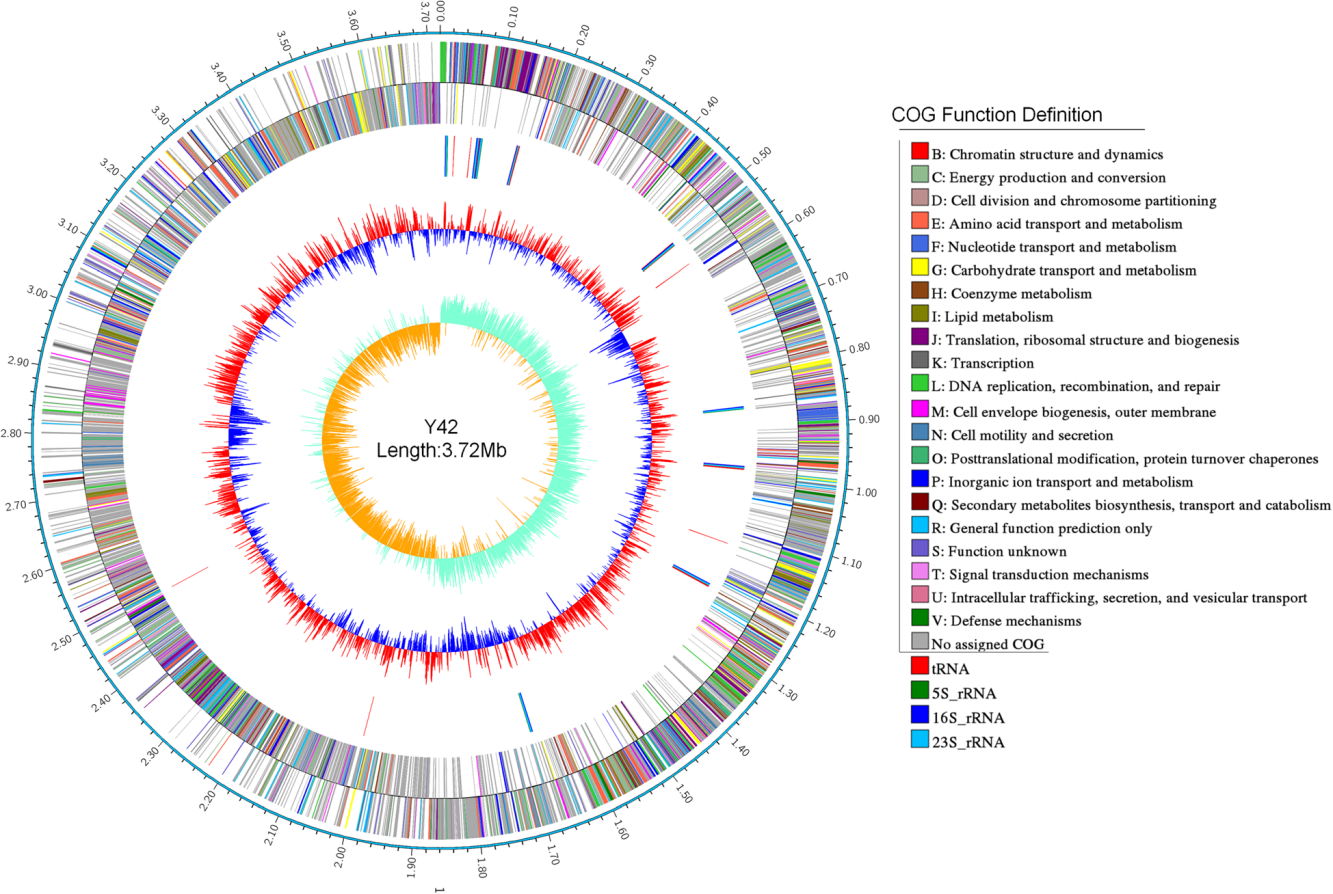
The genome map of *P. maritimus* strain Y42. The circles show the different descriptions of the content in megabases, from the outside to inward: outer two circles represent the predicted protein-coding sequences and CDS regions on the plus and minus strands, respectively. The colors represent COG functional classification. The circle 3 represent the predicted rRNA and tRNA. The 4th circle shows GC content and 5th circle exhibits the percent of GC-skew

The assembled genome of *P. maritimus* Y42 consisted of one circular DNA chromosome with a size of 3,718,896 bp and a GC content of 48.8% and three plasmids (329,482; 89,073; and 12,282 bp) (Table 3). Genome project information and genomic features are summarized in Table 4. From a total of 4155 genes, 3947 were annotated as predicted protein-coding sequences (CDS). In addition, the genome included 70 tRNA genes, 27 rRNA genes, 4 ncRNA genes, and 108 pseudogenes. Open reading frames (ORFs) were assigned into 23 functional categories under the Clusters of Orthologous Groups (COGs) and are represented in a circular genome map in Fig. 4. The COG distribution of genes is shown in Table 5. The genome map was visualized by the CG View server.

**Table 3:** Summary of genome: 1 chromosome and 3 plasmids

Label	Size (Mb)	GC%	INSDC identifier	RefSeq ID
Chromosome	3.72	48.8	CP019640.1	NZ_CP019640.1
Plasmid 1	0.329482	44.8	CP019641.1	NZ_CP019641.1
Plasmid 2	0.089073	43.6	CP019642.1	NZ_CP019642.1
Plasmid 3	0.012282	45	CP019643.1	NZ_CP019643.1

**Table 4:** Genome statistics of *P. maritimus* Y42

Attribute	Value	% of Total
Genome size (bp)	4,149,733	100
DNA coding (bp)	3,541,381	85.34
DNA G + C (bp)	2,005,184	48.32
DNA scaffolds	4	100
Total genes	4283	100
Protein coding genes	4172	97.41
RNA genes	111	2.59
Pseudo genes	108	
Genes in internal clusters	NA	
Genes with function prediction	3162	73.83
Genes assigned to COGs	2696	62.95
Genes with Pfam domains	3323	77.59
Genes with signal peptides	186	4.34
Genes with transmembrane helices	959	22.39
CRISPR repeats	NA

**Table 5:** Number of genes of *P. maritimus* Y42 with the general COG functional categories

Code	Value	% of total^a^	Description
J	225	7.34	Translation, ribosomal structure and biogenesis
A	0	0	RNA processing and modification
K	185	6.04	Transcription
L	117	3.82	Replication, recombination and repair
B	1	0.03	Chromatin structure and dynamics
D	36	1.17	Cell cycle control, Cell division, chromosome partitioning
V	71	2.32	Defense mechanisms
T	144	4.7	Signal transduction mechanisms
M	134	4.37	Cell wall/membrane biogenesis
N	47	1.53	Cell motility
U	33	1.08	Intracellular trafficking and secretion
O	118	3.85	Posttranslational modification, protein turnover, chaperones
C	183	5.97	Energy production and conversion
G	172	5.61	Carbohydrate transport and metabolism
E	297	9.69	Amino acid transport and metabolism
F	95	3.1	Nucleotide transport and metabolism
H	161	5.25	Coenzyme transport and metabolism
I	172	5.61	Lipid transport and metabolism
P	187	6.1	Inorganic ion transport and metabolism
Q	95	3.1	Secondary metabolites biosynthesis, transport and catabolism
R	325	10.6	General function prediction only
S	180	5.87	Function unknown
–	1587	37.05	Not in COGs

^a^The total is based on the total number of protein coding genes in the genome

## Insights from the genome sequence

Genome annotation predicted that many genes support the adaptability of strain Y42 to cold and crude oil-contaminated environments. Based on the COG analysis, the genes related to general function prediction only (R) and amino acid transport and metabolism (E) were relatively enriched over the other functional genes. The results indicate genome-wide selection pressure [33]. Moreover, the abundance of genes related to functions unknown (S) in strain Y42 suggested that the strain may possess many new genes.

**Fig. 5:**
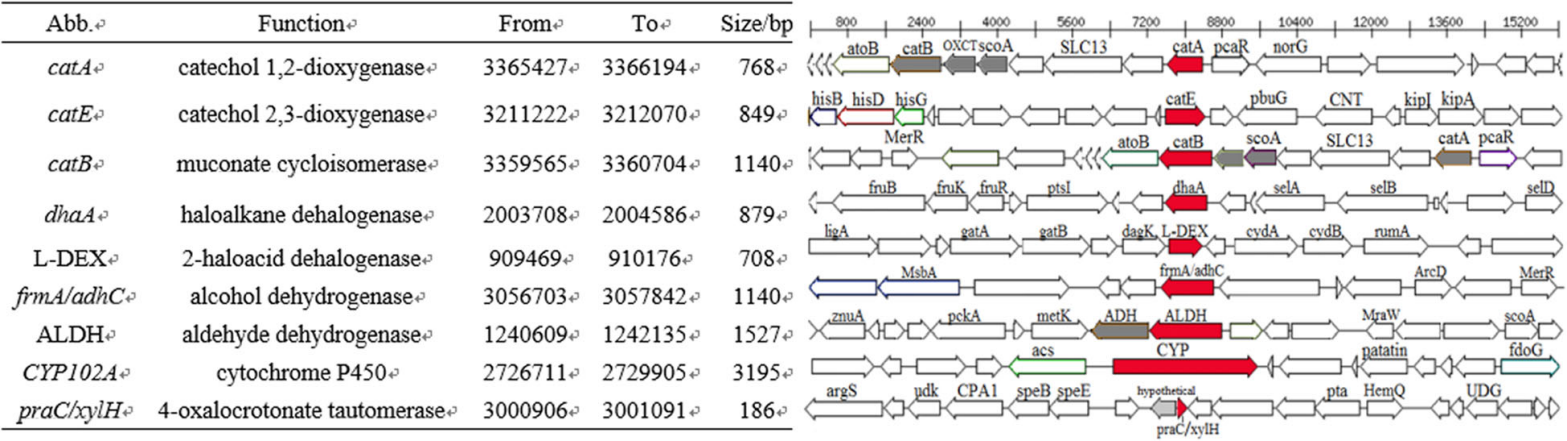
Gene clusters in the genome of *P. maritimus* strain Y42 encoding metabolic functions for oil degradation. The corresponding oil degradation related genes are red colored

Further analysis showed that many key oxygenase genes were located in the *P. maritimus* Y42 genome, including those of catechol 1,2-dioxygenase (*catA*), catechol 2,3-dioxygenase (*catE*), and cytochromes P450. In addition, dehalogenase-coding genes were also found in the chromosome; these genes were involved in numerous metabolic processes such as the degradation of chlorocyclohexane, chlorobenzene, chloroalkane and chloroalkene [34]. A total of 9 genes putatively encoding for crude oil metabolites were identified in this genome (Fig. 5). The existence of these oxygenase genes could regioselectively oxidize substrates, especially natural aromatic compounds, by transferring oxygen to the substrates and transforming non-reactive hydrocarbons into available hydrocarbons [35, 36]. However, genes responsible for *n*-alkane degradation, such as the *alkB* gene, which is considered as functional biomarker gene for alkane degradation [37–39], were not found in the genome of strain Y42. These results imply that the strain Y42 might have some novel genes that participate in the catabolism of *n*-alkane pollutants.

In addition, three cold shock proteins (WP_008296927.1, WP_026692369.1, WP_008298364.1.) were predicted, and these proteins were supposed to play important roles under low-temperature conditions [40]. In total, 238 genes were predicted to be involved in transport systems for aromatic compounds, amino acids, carbohydrates, lipids and inorganic ions. Among these genes, several osmoprotectant transport system (Opu) genes were identified to likely maintain the homeostasis of strain Y42. Furthermore, a large number of divalent cation transport and sulfate/phosphonate/nitrogen uptake systems guarantee the supply of nutrient elements for microbes in crude oil environments [41]. These genes were essential for strain Y42 to gain a competitive edge in oil-polluted soils.

## Conclusions

The strain Y42, as a potential new member of *Planococcus*, was isolated from a cold and crude oil-contaminated environment. A genomic analysis of strain Y42 provided the theoretical basis for the mechanism of oil degradation by bacteria. Genes involved in cold shock and transport systems point to the potential capacity of strain Y42 for soil bioremediation contaminated by aromatic compounds in cold environments. Genomic research on strain Y42 would also provide a blueprint for the application of bioremediation and recovery in cold oil-polluted environments.

## Abbreviation

CDSs: protein-coding sequences
COG: Clusters of Orthologous Groups categories
GC-MS: gas chromatography and mass spectrometric Detector
ORFs: open reading frames
